# Effect of nicotinamide mononucleotide on brain mitochondrial respiratory deficits in an Alzheimer’s disease-relevant murine model

**DOI:** 10.1186/s12883-015-0272-x

**Published:** 2015-03-01

**Authors:** Aaron N Long, Katrina Owens, Anna E Schlappal, Tibor Kristian, Paul S Fishman, Rosemary A Schuh

**Affiliations:** Research Service, VAMHCS, 10 North Greene Street, Baltimore, MD 21201 USA; Department of Neurology, University of Maryland, School of Medicine, Baltimore, MD 21201 USA; Program in Neuroscience and Cognitive Sciences, University of Maryland, College Park, MD 20742 USA; Department of Anesthesiology, Center for Shock, Trauma and Anesthesiology Research, School of Medicine, Baltimore, MD 21201 USA; Neurology Service, VAMHCS, Baltimore, MD 21201 USA; Present address: Department of Neurotrauma, Henry M. Jackson Foundation, Naval Medical Research Center, Silver Spring, MD 20910 USA

**Keywords:** Alzheimer’s disease, Mitochondria, Nicotinamide adenine dinucleotide, Nicotinamide mononucleotide, Neurodegeneration

## Abstract

**Background:**

Mitochondrial dysfunction is a hallmark of neurodegenerative diseases including Alzheimer’s disease (AD), with morphological and functional abnormalities limiting the electron transport chain and ATP production. A contributing factor of mitochondrial abnormalities is loss of nicotinamide adenine dinucleotide (NAD), an important cofactor in multiple metabolic reactions. Depletion of mitochondrial and consequently cellular NAD(H) levels by activated NAD glycohydrolases then culminates in bioenergetic failure and cell death. *De Novo* NAD^+^ synthesis from tryptophan requires a multi-step enzymatic reaction. Thus, an alternative strategy to maintain cellular NAD^+^ levels is to administer NAD^+^ precursors facilitating generation via a salvage pathway. We administered nicotinamide mononucleotide (NMN), an NAD^+^ precursor to APP_(swe)_/PS1_(ΔE9)_ double transgenic (AD-Tg) mice to assess amelioration of mitochondrial respiratory deficits. In addition to mitochondrial respiratory function, we examined levels of full-length mutant APP, NAD^+^-dependent substrates (SIRT1 and CD38) in homogenates and fission/fusion proteins (DRP1, OPA1 and MFN2) in mitochondria isolated from brain. To examine changes in mitochondrial morphology, bigenic mice possessing a fluorescent protein targeted to neuronal mitochondria (CaMK2a-mito/eYFP), were administered NMN.

**Methods:**

Mitochondrial oxygen consumption rates were examined in N2A neuroblastoma cells and non-synaptic brain mitochondria isolated from mice (3 months). Western blotting was utilized to assess APP, SIRT1, CD38, DRP1, OPA1 and MFN2 in brain of transgenic and non-transgenic mice (3–12 months). Mitochondrial morphology was assessed with confocal microscopy. One-way or two-way analysis of variance (ANOVA) and post-hoc Holm-Sidak method were used for statistical analyses of data. Student *t*-test was used for direct comparison of two groups.

**Results:**

We now demonstrate that mitochondrial respiratory function was restored in NMN-treated AD-Tg mice. Levels of SIRT1 and CD38 change with age and NMN treatment. Furthermore, we found a shift in dynamics from fission to fusion proteins in the NMN-treated mice.

**Conclusions:**

This is the first study to directly examine amelioration of NAD^+^ catabolism and changes in mitochondrial morphological dynamics in brain utilizing the immediate precursor NMN as a potential therapeutic compound. This might lead to well-defined physiologic abnormalities that can serve an important role in the validation of promising agents such as NMN that target NAD^+^ catabolism preserving mitochondrial function.

## Background

Alzheimer’s disease (AD) is the most common cause of dementia in the elderly, with aging being the most important risk factor [1]. Mitochondrial dysfunction is a hallmark of neurodegenerative diseases with morphological and functional abnormalities limiting the electron transport chain and adenosine triphosphate (ATP) production seen in AD [2].

Nicotinamide adenine dinucleotide (NAD) is a cofactor that is essential for many biological reactions in either its oxidized (NAD^+^) or reduced (NADH) forms [3]. NAD^+^ and NADH mediate transfer of hydrogens in oxidative and reductive metabolic reactions [4]. NAD^+^ is essential to many mitochondrial enzymatic reactions and appropriate cellular bioenergetic metabolism [5,6]. NAD^+^ levels naturally decline with aging [4]. In normal conditions, the loss of NAD^+^ inhibits cellular respiration, resulting in loss of mitochondrial ATP production and potentially cellular death [5]. NAD^+^ is used as a substrate by several NAD^+^ dependent enzymes including poly(ADP-ribose) polymerase 1 (PARP1), Sirtuin 1 (SIRT1), and ADP-ribosyl cyclase (CD38) [4,5,7,8].

Preventing NAD^+^ depletion and cellular energy deficits could be a therapeutic target for neurodegenerative diseases and act as a neuroprotective mechanism [7]. Four pathways can synthesize NAD^+^ in mammals. NAD^+^ can be synthesized from the salvage pathway (primary route) utilizing nicotinamide, nicotinic acid, nicotinamide riboside, or the *de novo* pathway using tryptophan [9]. Nicotinamide phosphoribosyltransferase (Nampt) helps transfer a phosphoribosyl residue to nicotinamide forming nicotinamide mononucleotide (NMN) [9]. NAD^+^ consists of NMN covalently bound to adenosine monophosphate (AMP) [4]. The enzyme NMN adenyltransferase (NMNAT) converts NMN to NAD^+^ in one step [4,9,10] making NMN a key precursor with possible therapeutic implications for increased NAD^+^ levels [11,12]. Further, NMN is more soluble than NAD^+^ in phosphate buffered saline (PBS) and is taken up more efficiently through the plasma membrane [9,13].

We have recently demonstrated in the well-studied AD chimeric APP_(swe)_/PS1_(ΔE9)_ mouse model, deficits in mitochondrial oxygen consumption rates (OCR) in both brain and muscle [14]. These deficits in OCR may have resulted from lack of sufficient NAD^+^ due to increased catabolism. Thus, in the present study we tested the hypothesis that increasing NAD^+^ availability by administering the precursor NMN would reverse mitochondrial OCR deficiencies in these AD disease-relevant animals. Mitochondrial respiration, calcium homeostasis and organelle transport have also been demonstrated to be influenced by mitochondrial morphology [15,16].

Fusion of two mitochondria causes an elongated morphology that can play a protective role in the nervous system, while fission allows proper distribution of mitochondria and is also used to remove damaged organelles [17]. Mitochondrial dynamics are the balance of fission and fusion, controlling the morphology, number, and function of mitochondria [17-19]. Abnormal changes in these dynamics have been linked with aging and several neurodegenerative diseases (e.g. AD, Huntington’s disease (HD), Parkinson’s disease (PD), multiple sclerosis (MS), and amyotrophic lateral sclerosis (ALS)). In these diseases mitochondrial morphology tends to shift towards increased fragmentation, indicating either an increase in fission or decreased fusion [19]. To examine changes in mitochondrial morphology, bigenic mice possessing a fluorescent protein targeted to neuronal mitochondria (CaMK2a-mito/eYFP), were administered NMN.

We demonstrate restoration of OCR in the NMN-treated AD double transgenic (AD-Tg) mice, indicating NAD^+^ levels were probably limiting. To further evaluate the basis of this effect we measured immunoreactivity of the NAD^+^-consuming proteins SIRT1 and CD38 and determined that they change with age as well as a function of NMN-treatment. Furthermore, we found a shift in dynamics from fission to fusion proteins in the NMN treated mice. This is the first study to directly examine amelioration of NAD^+^ catabolism and changes in mitochondrial morphological dynamics in AD mouse brain utilizing the immediate precursor NMN as a potential therapeutic compound.

## Methods

### Chemicals

All chemicals were purchased from Sigma-Aldrich (St Louis, MO) unless otherwise stated.

### Animals

#### Alzheimer’s disease-relevant mice

Double transgenic mice expressing a chimeric mouse/human amyloid precursor protein (APP) with the Swedish mutation (APP_swe_) and a mutant human presenilin 1 (PS1) with the delta E9 (PS1_ΔE9_) (strain # 005864) and wildtype C57BL/6 mice were purchased from the Jackson Laboratory, (Bar Harbor, ME). AD animals positive for the transgenes were identified by polymerase chain reaction (PCR) using genomic DNA, isolated from the tails (Qiagen, Valencia, CA) then processed as described previously [14].

#### CaMKIIα-tTA and pTRE-mito/eYFP mice

Transgenic mice expressing the tetracycline-controlled transactivator protein (tTA) under regulatory control of the calcium/calmodulin-dependent kinase II (CaMKII) promoter [20] and animals expressing pTRE-mito/eYFP [21] were purchased from the Jackson Laboratory. Bigenic mice positive for both CaMKII**α**-tTA and pTRE-mito/eYFP (CaMK2a-mito/eYFP) were generated by crossing these two strains. Male and female bigenic mice (2 months) were used in this study. CaMK2a-mito/eYFP bigenic mice were identified by PCR as described previously [21]. The University of Maryland School of Medicine Institutional Animal Use and Care Committee approved all procedures involving animal care, euthanasia and tissue collection.

### Nicotinamide mononucleotide (NMN) administration

APP_(swe)_/PS1_(ΔE9)_ and CaMK2a-mito/eYFP male and female mice (2 months) were administered NMN (100 mg/kg, Sigma N3501) in sterile PBS (200 μl) subcutaneously (in the loose skin around the neck and shoulder area) every other day for 28 days. Non-transgenic (NTG) and vehicle control animals were administered 200 μl sterile PBS subcutaneously every other day for 28 days. Subcutaneous administration was utilized based on pilot studies. No significant differences in weight or external characteristics (fur condition, energy, size etc.) were observed between NMN and vehicle treated mice (data not shown).

### N2A neuroblastoma cell culture conditions

Low passage mouse N2A hippocampal neuroblastomas (ATCC, Manassas, VA; 5,000/well) were seeded on V7 microplates (Seahorse Bioscience, North Billerica, MA) in proliferation media ((MEM, (ATCC), 10% fetal bovine serum, (Gibco, Grand Island, NY), 1% Pen-Strep, (Gibco)) and maintained in a humidified incubator at 37°C and 5% CO_2_. After 24 h, cultures were transiently transfected (see below). After a further 24 hours, the proliferation media was replaced with differentiation media (DM) consisting of MEM, 2% horse serum (Gibco) and 1% Pen-Strep. The cultures had media changes using DM 48 hours later and oxygen consumption rates were measured 24 hours later. All wells were critically examined under the microscope to ensure cell viability prior to performing experiments.

### Plasmid vector generation and transfection

The plasmid vector containing cDNA for a mitochondrially-targeted enhanced yellow fluorescent protein (eYFP), mutant APP_(swe)_ and mutant PS1_(ΔE9)_ described in [14] possesses a tetracycline response element thus requiring co-transfection with a tetracycline transactivator (TTA, Clontech). Co-transfection gives rise to cells possessing eYFP targeted to mitochondria and transgene-derived APP and PS1. N2A neuroblastomas were co-transfected with both constructs or TTA alone (control transfection) utilizing 1 μg of DNA/construct/well using the Magnetofection system (Oz Biosciences, San Diego, CA) with CombiMag plus lipofectamine (Invitrogen, Carlsbad, CA) according to the manufacturer’s protocol.

### Isolation of non-synaptic brain mitochondria

Twenty-four hours after the final NMN or vehicle injections, male and female APP_(swe)_/PS1_(ΔE9)_ or non-transgenic mice (3 months) were decapitated, forebrains rapidly removed and placed in ice-cold mannitol-sucrose (MS) buffer pH 7.4 (225 mM mannitol, 75 mM sucrose, 5 mM Hepes, 1 mg/ml fatty acid free BSA (Roche Diagnostics, Indianapolis, IN), 1 mM EGTA). Forebrains were homogenized with 10 strokes using a Potter-Elvehjem tissue grinder (Wheaton Science Products, Millville, NJ). The brain homogenates were further processed using the Percoll isolation method described by [22] and as used previously [14,23]. This method has been demonstrated to show a high level of mitochondrial purity by electron microscopy [23]. Protein concentrations were determined by the method described by [24] using BSA as standards. Aliquots of brain mitochondria and homogenate had protease inhibitors (Calbiochem, San Diego, CA) added prior to storage at −20°C for later Western blot analyses.

### N2A neuroblastoma cell respirometry

Prior to measurements, cultures were gently rinsed in pre-warmed (37°C) assay measurement buffer (MB) consisting of 120 mM NaCl, 3.5 mM KCl, 1.3 mM CaCl_2_, 0.4 mM KH_2_PO_4_, 1 mM MgCl_2_, 5 mM HEPES (pH 7.4) supplemented with 2.5 mM D-glucose. The cells were then placed in an unbuffered, humidified incubator at 37°C for 2 hours to allow temperature and pH equilibration. Cells were visually inspected prior to and after MB addition then loaded onto the Seahorse XF24-3 flux analyzer (Seahorse Bioscience). After an equilibration step, basal oxygen consumption rates (OCR, pMoles/min) were recorded using 3-min mix, 2-min wait, and 3-min measure (looped 3 times) cycles prior to injection of oligomycin to inhibit the ATP synthase. Three more measurement loops were recorded prior to injection of carbonyl cyanide p-(trifluoromethoxy)phenylhydrazone (FCCP) to induce maximal oxygen consumption. Following recording of 3 more measurement loops, pyruvate was injected to determine if maximal oxygen consumption following FCCP addition was substrate limited. Antimycin A (inhibitor of mitochondrial respiration) was injected after 3 measurement loops to assess non-mitochondrial OCR. Two measurement loops were recorded after antimycin A injection then the experiment was terminated. The injectates prepared in MB (75 μl volumes) were preloaded, then sequentially injected as indicated through ports in the XF24 calibration cartridge to final concentrations of 1 μg/ml oligomycin, 1 μM FCCP, 10 mM pyruvate, and 1 μM antimycin A. Each plate had a subset of cells incubated with 10 mM nicotinamide adenine dinucleotide (NAD^+^, during the DM pre-incubation) prior to measurements.

### Brain mitochondrial respirometry

Following calibration of the Seahorse XF24-3 flux analyzer (Seahorse Bioscience), the final non-synaptic mitochondrial pellets from individual mouse brains were resuspended in MAS1 buffer [25] and 5 μg protein as determined above [24] loaded into each of 20 wells of an XF24 V7 cell culture plate (Seahorse Bioscience). The plates were centrifuged at 1,600 × *g* at 4°C for 5 min. MAS1 buffer with 5 mM L-malate plus 5 mM sodium pyruvate (freshly made in MAS1 buffer) was gently added to the wells and the plates immediately loaded onto the instrument and oxygen consumption measurements were recorded as previously described [14]. All measurements were performed at 37°C.

### Immunoblotting

Proteins as determined by [24] from brain homogenates or non-synaptic mitochondria (50 μg) of APP_(swe)_/PS1_(ΔE9)_ and their non-transgenic litter mates (3 months) were resolved using sodium dodecyl sulfate polyacrylamide gel electrophoresis (SDS-PAGE) on precast Mini-Protean TGX any KD gels (Bio-Rad, Hercules, CA) and transferred to a polyvinylidene difluoride membrane using a Trans-Blot Turbo transfer system (Bio-Rad). Immunoblotting was performed according to Li-Cor Biosciences (Lincoln, NE) protocol. Briefly, nonspecific sites were blocked in non-mammalian blocking buffer (Li-Cor Biosciences). After blocking, the membranes were incubated with primary antibodies to Beta amyloid 1–16 (6E10, 1:1,000; Covance); Histone deacetylase sirtuin 1 (SIRT1, 1:500; Millipore); NAD^+^ glycohydrolase CD38 (CD38; 1:2,000; R&D, Minneapolis, MN); Dynamin-related protein 1 (DRP1, 1:1,000; BD Biosciences, San Jose, CA); Phospho-DRP1 (P^616^-DRP1, 1:1,000; Cell Signaling Technology, Danvers, MA); Mitofusin 2 (MFN2, 1:1,000; Abcam, Cambridge, MA); Optic atrophy protein (OPA1, 1:1,000; BD Bioscience); Glyceraldehyde 3-phosphate dehydrogenase (GAPDH; 1:14,000; Cell Signaling Technology); Voltage dependent anion channel (VDAC, 1:1,000; (rabbit); Cell Signaling Technology); VDAC (mouse; 1:1,000; Mitosciences (Eugene, OR); β-Actin (1:10,000; Sigma) at 4°C overnight. After 4 × 5 min washes in phosphate buffered saline (PBS) with 0.1% tween-20 (PBST), the membranes were incubated in the appropriate infrared (IR) fluorophore conjugated secondary antibody (Li-Cor Biosciences) for 30 min in the dark. Following PBST washes, the IR signal was captured on an Odyssey infrared imaging system (Li-Cor Biosciences) and stored as a digital image. VDAC was utilized as the loading control for mitochondria and GAPDH or β-Actin for brain homogenate to ensure equal loading.

### Histology

Male and female CaMK2a-mito/eYFP, (3 months) were perfusion-fixed under deep anesthesia then processed as previously described [26].

### Laser scanning confocal microscopy and quantitation of mitochondrial morphology

Forty μm-thick coronal brain tissue sections from CaMK2a-mito/eYFP mice (3 months) were washed with potassium phosphate buffered saline (KPBS) then mounted and coverslipped using Vectashield Hard set (Vector, Burlingame, CA) mounting media. Mitochondria in brain sections from CA1 hippocampal sub-regions were imaged utilizing a Zeiss LSM 510 laser scanning confocal microscope using a Plan-Apochromat 63x/1.4 oil lens. Single planes of 1024 × 1024 pixels were recorded at 1.0 – 1.5 Airy unit pinhole every 0.2 μm z-spacing throughout the entire tissue section as previously described [26] with modifications. Specifically, z-stack images were obtained from the striatum oriens of the CA1 sub-region. A 488 nm laser was used to visualize eYFP. Four z-stack images were taken per mouse brain. Recorded images were analyzed with Volocity software (Perkin Elmer, Waltham, MA). Quantification of mitochondrial morphology utilizing Volocity software was performed as previously described [26]. The following equation was used to determine 3D shape factor (ratio of the surface area of a sphere (with the same volume as the given object) to the surface area of the object): V_0_ = volume of object; A_0_ = area of object

$$ 3\mathrm{D}\ \mathrm{Shape}\ \mathrm{Factor}=\frac{\pi^{1/3}{\left(6{V}_0\right)}^{2/3}}{A_0} $$

### Statistical analysis

Data are expressed as means ± SE, and the comparisons between experimental groups were made with SPSS statistical software (SPSS, Inc., Chicago, IL) using analysis of variance (ANOVA). Posthoc Holm-Sidak method was used for all pairwise comparisons after ANOVA tests. Student *t*-test was used when direct comparison of two groups were analyzed (volocity data). Significance was assumed at p < 0.05.

## Results

### Exogenous NAD^+^ reverses deficient oxygen consumption rates (OCR) in a cell-based model of amyloid beta toxicity

**Figure 1:**
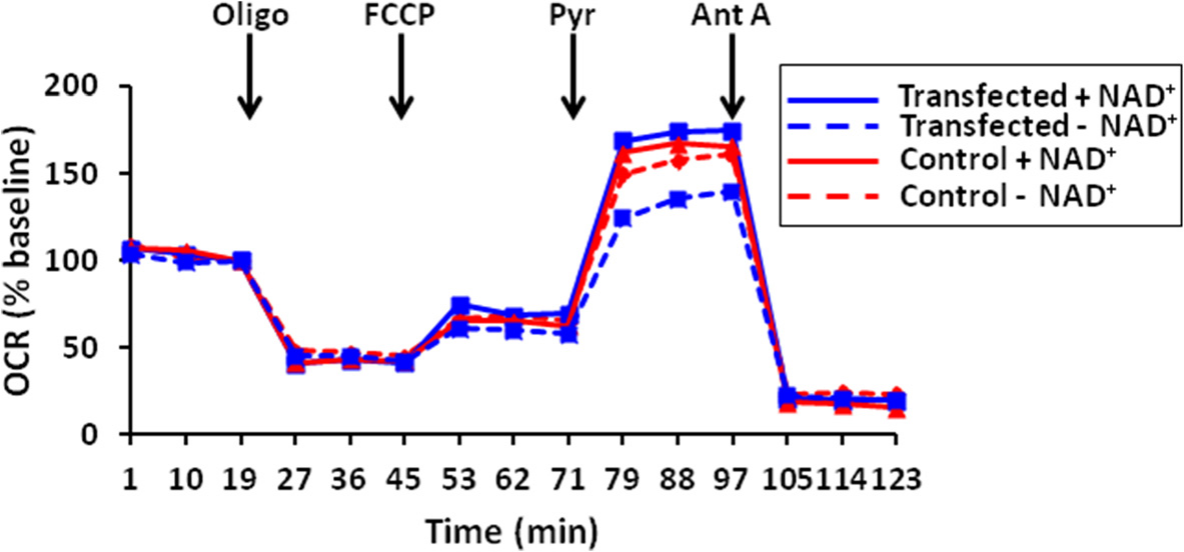
**OCR in APP**_**(swe)**_ **-overexpressing N2A hippocampal neuroblastoma cells (blue lines) and control transfected cells (red lines).** Baseline-normalized oxygen consumption rates (OCR) of N2A neuroblastoma cells exposed to successive additions of mitochondrial respiratory modulators (arrows) are shown. Cells received 1 μg/ml oligomycin (Oligo), 1 μM FCCP, 10 mM pyruvate (Pyr), and 1 μM antimycin A (Ant A). Subsets of the cells (solid lines) were pre-incubated with 10 mM nicotinamide adenine dinucleotide (NAD^+^) prior to measurements while control cells (dashed lines) had no exogenous NAD^+^. Rates are normalized to the third baseline measurement point for n = 3–5 replicates per group from 2 separate cultures.

N2A hippocampal neuroblastoma cells were transiently co-transfected with constructs containing mutant APP_(swe)_/PS1_(ΔE9)_ and a tetracycline transactivator (TTA) as a cell-based model of effects of mutant amyloid toxicity. Co-transfected (transfected) or TTA-transfected (control) N2A cells were pre-incubated ± nicotinamide adenine dinucleotide (NAD^+^) and oxygen consumption rates (OCR) measured. Transfected N2A cells (no exogenous NAD^+^; blue dashed line) had decreased maximal OCR when challenged with uncoupler carbonyl cyanide p-(trifluoromethoxy) phenylhydrazone (FCCP) and pyruvate, compared to control cells + NAD^+^ (24% decrease; Figure 1, red solid line); or control cells without exogenous NAD^+^ addition (18% decrease; Figure 1, red dashed line). This OCR deficit (Figure 1, blue dashed line) could be ameliorated if exogenous NAD^+^ was present (27% increase; Figure 1, blue solid line). Control N2A cultures had similar OCR regardless of NAD^+^ addition (Figure 1, red solid versus red dashed line). These experiments suggest that deficiencies in NAD^+^ levels could play a role in mitochondrial respiratory dysfunction in transgenic mice expressing this mutation *in vivo* [14] and could be corrected with added NAD^+^.

### Decreased full-length mutant human Amyloid Precursor protein (APP) levels in brain of NMN treated transgenic mice

**Figure 2:**
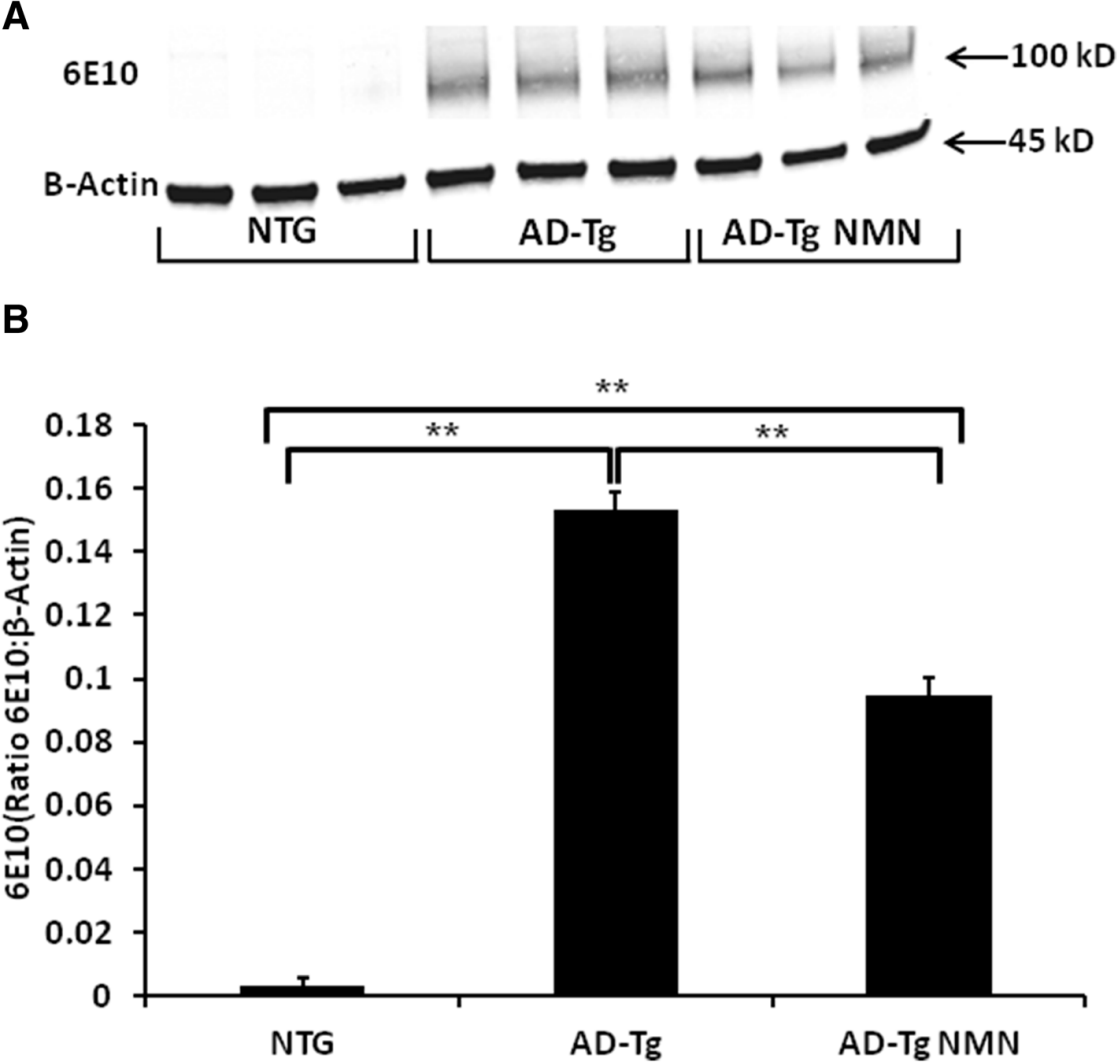
**Full-length, transgene-derived amyloid precursor protein (APP) levels in brain homogenates following NMN treatment. (A)** Representative Western blots of homogenates isolated from the brains of AD-Tg and non-transgenic (NTG) mice (3 months) probed with 6E10 antibody. **(B)** Transgene-derived full-length APP (~106kD) is observed in AD-Tg mice with negligible levels in the NTG mice. Full-length APP levels (ratio of APP:β-Actin) are significantly decreased in NMN-treated transgenic (AD-Tg NMN) mice compared to AD-Tg vehicle-treated. Data are presented as the average full-length APP ± SE. N = 6 separate animals per group. *p < 0.05.

Forebrain homogenates were assessed for relative transgene-derived full-length APP expression in both APP_(swe)_/PS1_(ΔE9)_ transgenic (AD-Tg) and non-transgenic (NTG) mice (3 months). There were significantly (p < 0.05) increased mutant full-length APP levels in the brain homogenates from the AD-Tg mice regardless of treatment when compared to non-transgenic littermates. There was a significant (p ≤ 0.05) decrease (38%) in full-length mutant APP levels in brain homogenates of AD-Tg mice treated with NMN as compared to AD-Tg vehicle-treated mice (Figure 2).

### Oxygen consumption deficits in brain mitochondria isolated from AD-Tg mice are reversed by nicotinamide mononucleotide (NMN)

**Figure 3:**
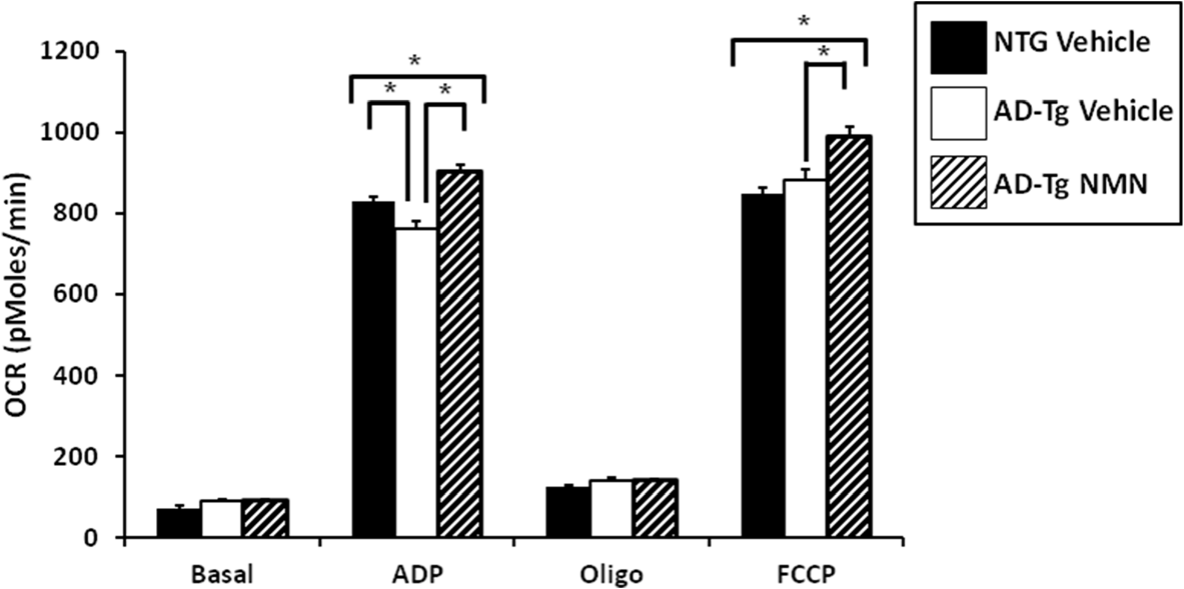
**OCR deficits in non-synaptic brain mitochondria from mutant APP expressing mice following NMN treatment.** Average OCR (pMoles/min) in non-synaptic mitochondria isolated from APP_(swe)_/PS1_(ΔE9)_ (AD-Tg) and non-transgenic (NTG) mouse (3 months) brain. Solid black bar (NTG vehicle control), white bar (AD-Tg vehicle control) and striped bar (AD-Tg NMN). Data are presented as the average OCR ± SE. N = 3–5 separate animals per group. *p < 0.01.

In a previous study, we determined that brain mitochondrial ADP-stimulated OCR were significantly deficient in 3 months old male AD-Tg mice as compared to their non-transgenic littermates [14]. NAD^+^ as a cofactor for tricarboxylic acid (TCA) cycle and Complex I enzymatic reactions and deficiencies give rise to bioenergetic dysfunction. The NAD^+^ precursor nicotinamide mononucleotide (NMN) inhibits NAD^+^ degradation as well as enhancing its synthesis [27]. Therefore, to examine the potential relationship between mitochondrial respiratory dysfunction and NAD^+^ catabolism, AD-Tg and non-transgenic (NTG) mice (2 months) were given NMN or phosphate buffered saline (PBS, vehicle). Non-synaptic mitochondria were then isolated from the forebrains of male and female (3 months) AD-Tg mice and their NTG littermates. There were no significant differences in basal OCR between the transgenic and non-transgenic mice regardless of NMN treatment (Figure 3, Basal). However, following addition of ADP to initiate State 3 respiration, the AD-Tg vehicle-treated mice had significantly lower OCR (p < 0.01) compared to the NTG animals (Figure 3, ADP) recapitulating our previous study [14]. The AD-Tg vehicle-treated mice were significantly deficient (p < 0.01) in State 3 respiration as compared to the transgenic mice that received NMN treatment (Figure 3, ADP). Interestingly, the AD-Tg NMN-treated mice had significantly increased (p < 0.01) OCR as compared to the non-transgenic animals (Figure 3, ADP). Following oligomycin addition reducing the rate of O_2_ consumption to that of State 4_°_ respiration, there was no significant difference in OCR between the transgenic and non-transgenic mice regardless of treatment group (Figure 3, Oligo). Although, following addition of FCCP to assess maximal OCR there was no significant difference between NTG vehicle and AD-Tg vehicle-treated mice, the AD-Tg NMN-treated mice had significantly increased (p < 0.01) OCR as compared to both the transgenic vehicle-treated and non-transgenic animals (Figure 3, FCCP).

### Histone deacetylase sirtuin1 (SIRT1) and CD38 protein levels in brain homogenates

**Figure 4:**
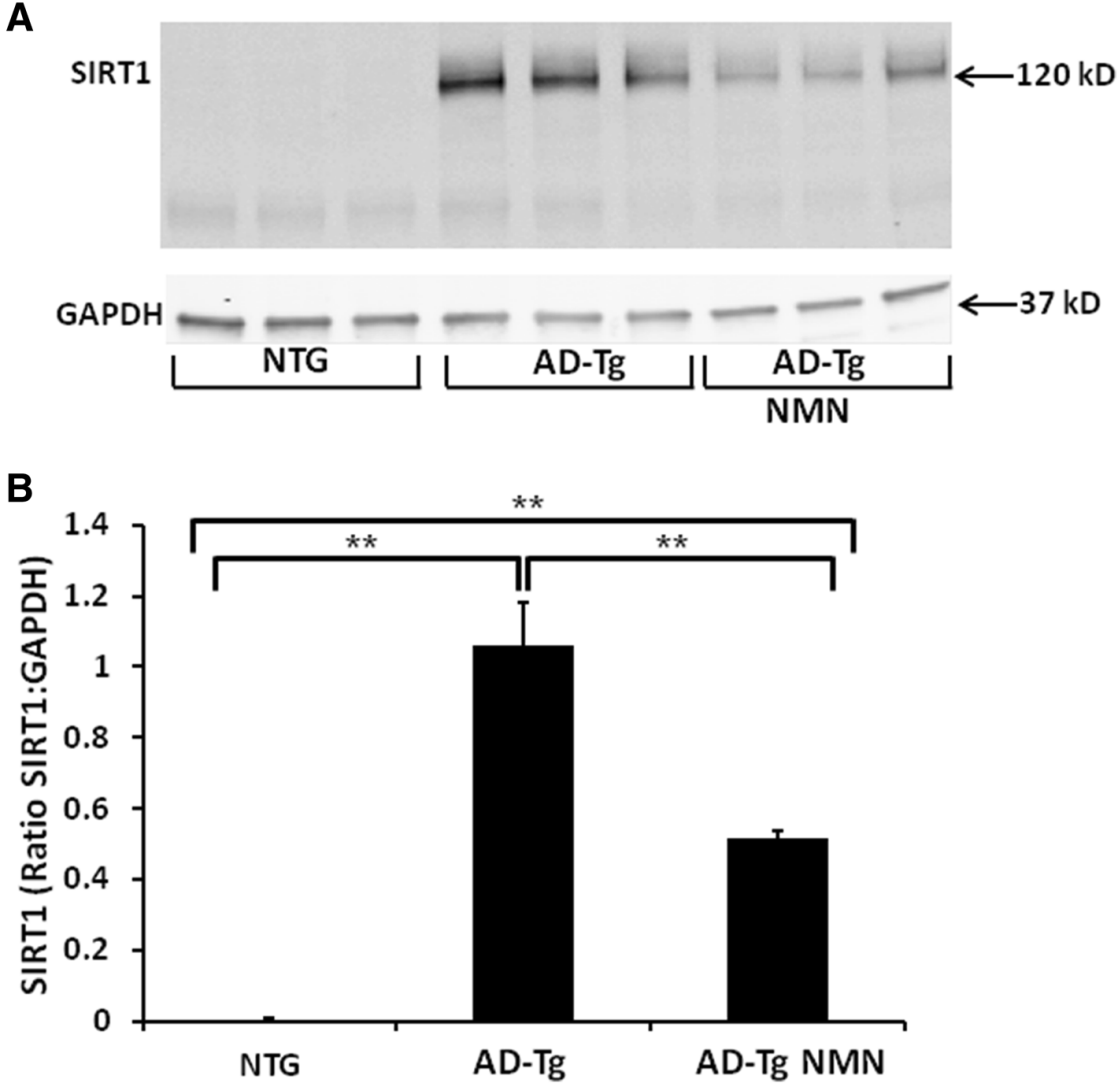
**Immunoreactivity of SIRT1 in homogenates isolated from NTG and AD-Tg mouse brain following NMN treatment.** Each lane represents a different animal. **(A)** Representative Western blot images. **(B)** Significantly increased SIRT1 levels (ratio SIRT1:GAPDH) are observed in AD-Tg (vehicle) homogenates compared to NTG vehicle-treated animals. These levels are significantly reduced (~49%) by the addition of NMN in AD-Tg mice although they remain significantly elevated. NTG SIRT1 levels normalized to GAPDH = 0.004 as presented in **(B)**. Data are presented as the average SIRT1 ± SE. N = 6 separate animals per group. **p ≤ 0.05.

NAD^+^ is utilized as an important cofactor in many metabolic reactions [28] including oxidative phosphorylation and enzymatic reactions of the TCA cycle [5], and as a substrate for enzymes including histone deacetylase sirtuin 1 (SIRT1), poly(ADP-ribose) polymerase 1 (PARP1) and NAD^+^ glycohydrolase CD38. We assessed brain homogenates from AD-Tg and non-transgenic NMN- and vehicle-treated mice to determine if NAD^+^ catabolism was due to increased SIRT1 levels. There was a significant increase (p < 0.05) in SIRT1 immunoreactivity in brain homogenates from AD-Tg mice when compared to both NTG animals as well as AD-Tg animals pre-treated with NMN (Figure 4). Although the AD-Tg mice pre-treated with NMN had lower SIRT1 immunoreactivity compared to non-NMN treated AD-Tg mice, these levels were still significantly elevated (p ≤ 0.05) as compared to NTG animals (Figure 4).

**Figure 5:**
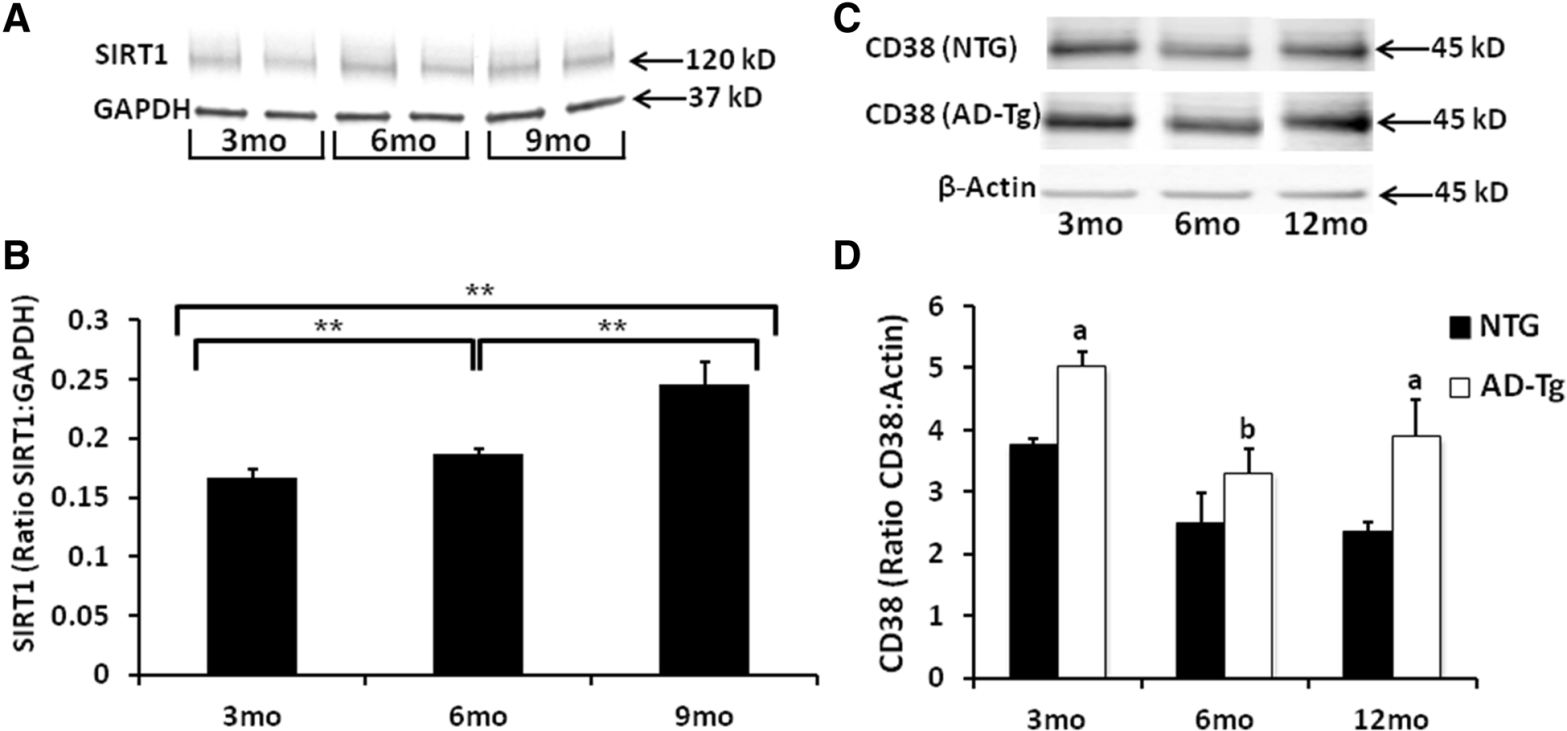
**Altered SIRT1 and CD38 immunoreactivity with age.** Representative Western blot images for SIRT1 **(A)** and CD38 **(C)** in brain homogenates isolated from NTG and AD-Tg mice (3–12 months). **(B)** Significantly increased SIRT1 levels (ratio SIRT1:GAPDH) are observed in AD-Tg homogenates of 6 months compared to 3 months; and 9 months compared to both 3 and 6 months. **(D)** Significant increases in CD38 levels are observed at 3 and 12 months in AD-Tg homogenates compared to NTG mice. Significant decrease in CD38 levels are observed at 6 months in AD-Tg homogenates compared to 3 months AD-Tg mice. Data are presented as the average SIRT1 or CD38 ± SE. N = 3 separate animals per age group. **p < 0.05; *p < 0.01; a: AD-Tg significantly different from NTG; b: 6 months AD-Tg significantly different from 3 months AD-Tg.

SIRT1 immunoreactivity in brain homogenates was examined to assess potential additive effects of AD transgenes in older animals (3–9 months). There was significantly (p < 0.05) increased SIRT1 immunoreactivity in the 9 months AD-Tg mice as compared with both the 3 and 6 months animals (Figure 5A, B). Further, the 6 months AD-Tg SIRT1 levels were also significantly (p ≤ 0.05) elevated compared to the 3 months AD-Tg mice (Figure 5A, B). As described above, an alternative NAD^+^ consumer is the NAD^+^ glycohydrolase CD38 (CD38). We therefore also probed brain homogenates from both NTG and AD-Tg mice aged 3–12 months utilizing Western blots. There was a significant increase (p < 0.05) in CD38 immunoreactivity in the 3 and 12 months AD-Tg mice as compared to age-matched NTG animals (Figure 5C,D). There was a significant decrease (p < 0.05) in CD38 immunoreactivity between 3 and 6 months AD-Tg mice (Figure 5C,D). There was no significant difference in CD38 immunoreactivity in NTG mice across the age groups examined. Although there was an overall significant difference in CD38 levels comparing genotypes (p < 0.01) or age (p < 0.01), there was no genotype x age interaction (Figure 5C, D).

### Morphological analyses of brain mitochondria from CaMK2a-mito/eYFP mice

To begin examining potential explanations for the ameliorative benefits following *in vivo* addition of the NAD^+^ precursor NMN, we examined morphological parameters in brain tissue of mice (3 months) possessing mitochondrial-targeted fluorescent proteins in neurons (CaMK2a-mito/eYFP mice). Mitochondrial morphology has been demonstrated to influence respiration rates, calcium homeostasis and organelle transport [15,16].

**Figure 6:**
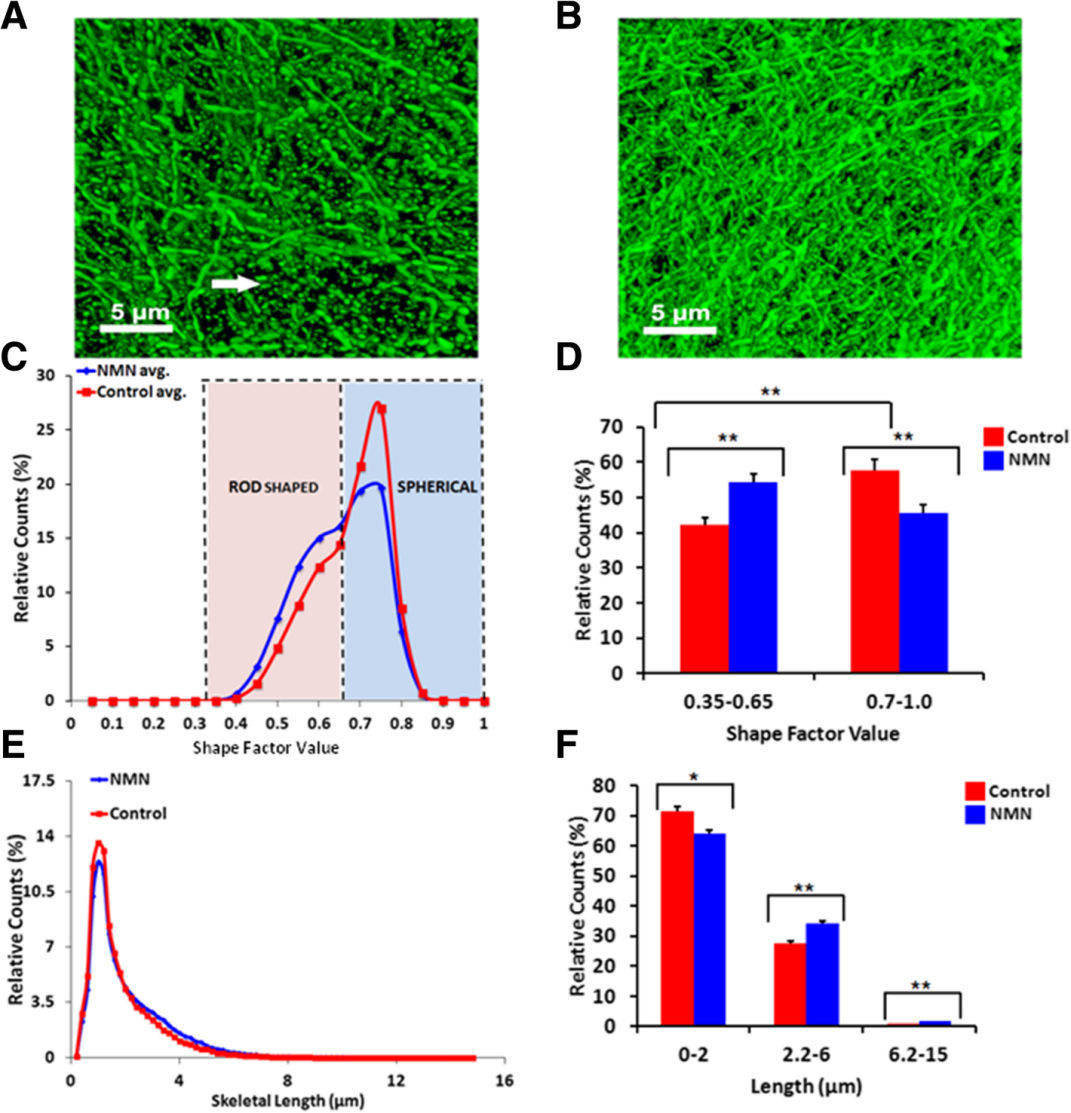
**Changes in mitochondrial dynamics following NMN treatment in CA1 hippocampal neurons from CAMK-eYFP mice.** Representative confocal images of control **(A)** and NMN-treated **(B)** neuronal mitochondria in the CA1 hippocampal sub-region. The fluorescent mitochondria appear more fragmented (see white arrows) in the control image compared to NMN treatment that clearly demonstrates more elongated mitochondria. Graphs **(C, D)** show the relative shape distribution and the relative mitochondrial length distribution **(E, F)** of different mitochondrial populations. Scale bar in panels **(A)** and **(B)** represents 5 μm. **p < 0.05; *p < 0.01.

Brain mitochondrial respiratory rates in CaMK2a-mito/eYFP mice have been previously determined to be similar to the rates in wildtype mice [21] and therefore can be utilized as an important tool to examine morphology. Following the same protocol for NMN treatment as described in the AD animals above, CaMK2a-mito/eYFP mice given vehicle had more fragmented neuronal mitochondria in the CA1 region of the hippocampus when compared to CaMK2a-mito/eYFP animals given NMN (Figure 6A). The CaMK2a-mito/eYFP mice given NMN had far less fragmented mitochondria, but had longer neuronal mitochondria in the CA1 region (Figure 6B).

We next examined the shape of fluorescent neuronal mitochondria in the CaMK2a-mito/eYFP mice. Shape factor is a numerical value indicating how similar a 3D shape is to a perfect sphere. Thus, a shape factor value of 1 was considered to be completely spherical whereas the closer to zero was considered to be rod-shaped. Figure 6C diagrams the relative counts comprising the shape factor value of neuronal mitochondria from NMN-treated versus vehicle-treated mice. When the shape of neuronal mitochondria in the CA1 hippocampal region were examined, there was a significant (p < 0.05) increase in rod-shaped mitochondria in NMN-treated mice compared to vehicle-treated animals (Figure 6D). There was a reciprocal decrease in spherical-shaped mitochondria in these same regions in NMN-treated mice as compared to vehicle-treated animals that were significantly (p < 0.05) altered (Figure 6D). Interestingly, there was a significant (p < 0.05) difference between rod and spherical-shaped mitochondria in the vehicle-treated mice but no significant shape difference in the NMN-treated animals (Figure 6D). Following quantification of fluorescent neuronal mitochondria in the CA1 hippocampal regions, there was a significant difference in mitochondrial skeletal length when comparing NMN treated and vehicle treated CaMK2a-mito/eYFP mice (Figure 6E, F). The relative counts for skeletal length were sub-divided into “spherical” (0–2 μm), “rod shaped” (2.2-6 μm) and “tubular” (6.2-15 μm). The control mice had significantly (p < 0.05) increased “spherical” length mitochondria compared to the NMN treated animals (Figure 6F). Conversely, the control mice had significantly decreased “rod shaped” (p < 0.01) and “tubular” (p ≤ 0.001) length mitochondria compared to NMN treated animals (Figure 6F). There were no significant differences in the relative counts for total volume or total surface area between treatment groups (data not shown). Further, the total number of mitochondria approached significance with a trend toward less mitochondria in NMN-treated mice (p = 0.066), as compared to vehicle-treated animals (data not shown). Together, these data suggest that NMN treatment gives rise to longer mitochondria in the hippocampal sub-region examined, likely through increased fusion and/or decreased fission.

### Altered Drp1 phosphorylation mediates mitochondrial fragmentation in AD-Tg mice

Mitochondrial morphology is modulated by a dynamic balance between fragmentation (fission) and formation of tubular elongated structures (fusion) (reviewed in [29,30]. Fission and fusion protein immunoreactivity was therefore examined in mitochondria isolated from the NTG and AD-Tg mice above that were given NMN or vehicle.

**Figure 7:**
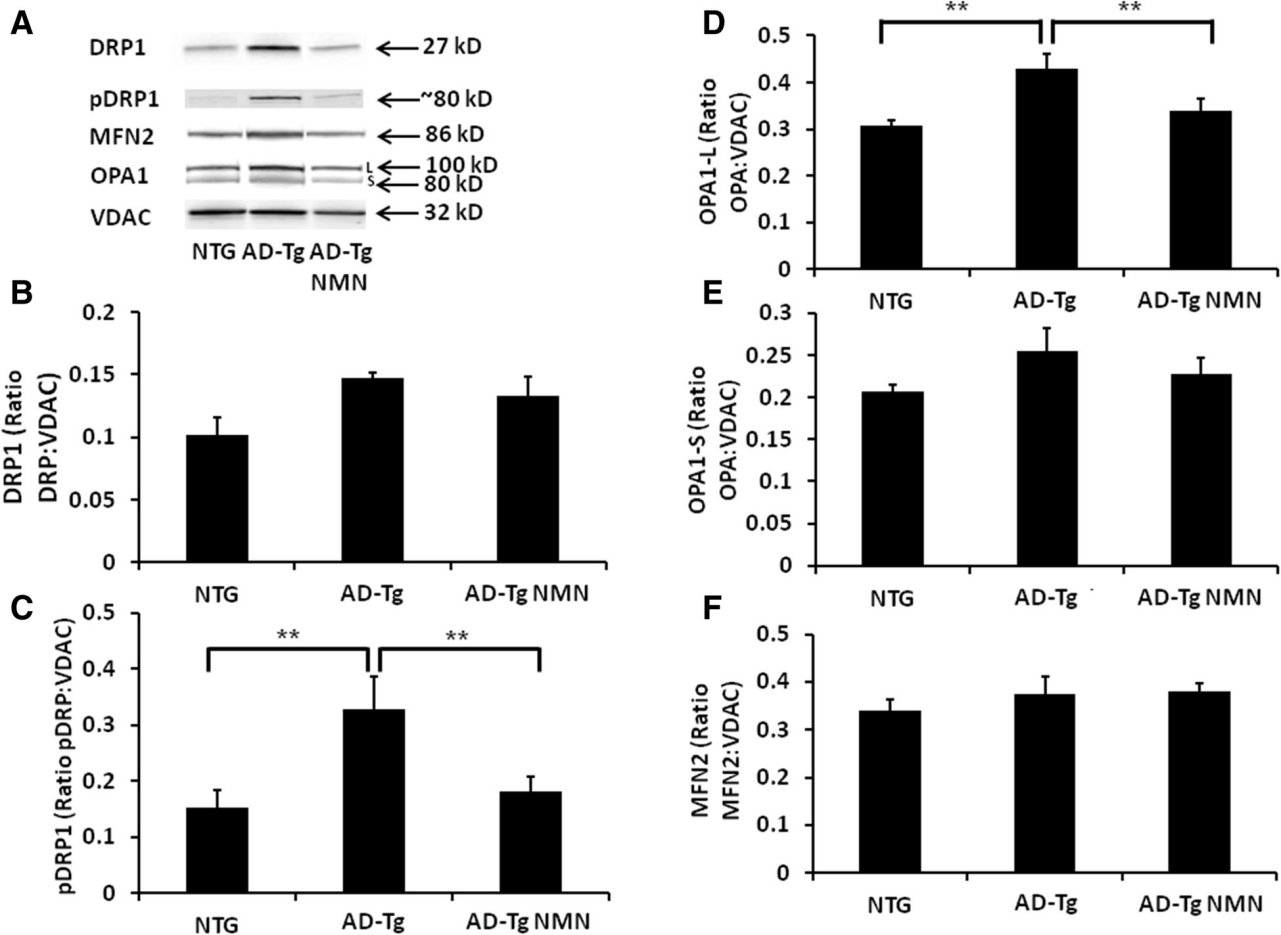
**Immunoreactivity of fission/fusion proteins in brain mitochondria isolated from mouse brain following NMN treatment. (A)** Representative Western blot images of fission (DRP1 and phosphorylated form P^616^-DRP1) and fusion (OPA1 and MFN2) proteins. **(B-F)** Graphs showing quantification of the Western blots in (A). The OPA1 oligomeric complex comprises the long (L) fusogenic form and the short (S) form [32]. Voltage-dependent anion channel (VDAC) was utilized as a loading control. Data are presented as the average DRP, P^616^-DRP1, OPA1_L_ OPA1_S,_ MFN2 ± SE. N = 5–7 separate animals per group. **p < 0.05.

The dynamin-related protein 1 (DRP1) with mitochondrial fission 1 protein (Fis1) control mitochondrial fragmentation. When DRP1 immunoreactivity was assessed in isolated brain mitochondria, there was a trend toward an increase in DRP1 immunoreactivity in AD-Tg mice as compared to NTG animals (p = 0.072) that was not changed in the NMN-treated cohort (Figure 7A, B). When DRP1 is phosphorylated at a specific serine residue (Ser616, P^616^-DRP1), stabilization of the outer membrane of cytoplasmic DRP1 occurs [31]. There was a significant (p < 0.05) increase in P^616^-DRP1 immunoreactivity in mitochondria isolated from AD-Tg mice as compared to NTG animals (Figure 7A, C). The AD-Tg mice treated with NMN had significantly (p < 0.05) decreased P^616^-DRP1 immunoreactivity as compared to AD-Tg vehicle-treated animals (Figure 7A, C). There was no significant difference in mitochondrial P^616^-DRP1 immunoreactivity between NTG animals and AD-Tg mice treated with NMN (Figure 7A, C).

### OPA1 and MFN2 immunoreactivity are differentially altered in AD-Tg mice

We next examined immunoreactivity in these samples for mitofusin 2 (MFN2) and optic atrophy protein (OPA1), both associated with mediation of mitochondrial fusion.

Optic atrophy 1 (OPA1) is a dynamin-related GTPase that resides in the mitochondrial inner membrane (IMM) promoting regulation of mitochondrial fusion [32]. OPA1 as an oligomeric complex in healthy mitochondria maintains cristae integrity. The oligomeric complex comprises a heterotrimer formed by integral IMM (long form ~100 kD; OPA1-L) and soluble inter-membrane space (IMS, short form ~80 kD; OPA1-S) forms of OPA1. There was a significant (p < 0.05) increase in OPA1-L immunoreactivity in AD-Tg mice compared to both NTG animals and AD-Tg mice that received NMN (Figure 7A, D). There was no significant difference in OPA1-L immunoreactivity between the NTG and AD-Tg mice that received NMN (Figure 7A, D). There was no significant difference in immunoreactivity across groups for the OPA1-S form (Figure 7A, E).

Mitofusin 2 (MFN2) also a GTPase is involved in fusion of the mitochondrial outer membrane (OMM). There were no significant differences in MFN2 levels across groups (Figure 7A, F). Taken together, these data demonstrate that the AD-Tg mice have increased mitochondrial fragmentation compared to NTG animals and also mitochondrial fusion occurring which is ameliorated in the transgenic mice given exogenous NMN.

## Discussion

Alzheimer’s disease, along with other neurodegenerative diseases, has a complex multifactorial pathology with known mitochondrial deficits. This study is the first to directly examine the amelioration of NAD^+^ catabolism and changes in mitochondrial morphological dynamics in AD mouse brain utilizing the immediate precursor nicotinamide mononucleotide (NMN) as a potential therapeutic compound. At three months, the well-studied AD chimeric APP_(swe)_/PS1_(ΔE9)_ (AD-Tg) mouse model have mitochondrial oxygen consumption rate (OCR) deficits [14] that precede amyloid deposition and plaque formation in brain [33]. In the present study, deficiencies in OCR were successfully reversed using the same murine AD model administered NMN. In addition, NMN reduced levels of full length mutant APP in the AD-Tg mice. Further, the effects of NMN on normal mitochondrial morphology were examined using mice possessing fluorescent proteins targeted to neuronal mitochondria (CaMK2a-mito/eYFP) and demonstrate mitochondrial elongation and decreased fragmentation in NMN treated animals.

Nicotinamide (NAM) administration has been demonstrated to cross the blood–brain barrier and be converted to NAD^+^, thus increasing cellular NAD^+^ levels in the brain [34]. Further, NMN has been shown to be highly enriched in mitochondria by sub-cellular fractionation studies suggesting intramitochondrial NAD^+^ synthesis [35] and may inhibit CD38 NAD^+^ glycohydrolase activity [27,36]. Several disease/injury model studies have previously utilized NMN as a therapeutic to ameliorate NAD^+^ deficiencies.

Yoshino et al. [37] reversed NAD^+^ deficits in a diabetic mouse model with intraperitoneal (IP) injections of NMN (500 mg/kg body weight/day for 7 days) a higher total dosage compared to the present study. In a separate study, NAD^+^ levels were increased within 30 minutes after NMN administration (IP, 500 mg/kg body weight) directly following ischemia and reperfusion in mouse heart [9]. Further, NMN prevented decreases in NAD^+^ if injected 30 minutes prior to the insult [9]. In a cell culture model of Parkinson’s disease consisting of rotenone treated PC12 cells, NMN intervention reduced apoptosis and restored intracellular levels of NAD^+^ and ATP [11]. In the present study, we used early low dose NMN administration beginning at two months of age to prevent the OCR deficits seen previously in three months old AD-Tg mice [14].

Studies of NAD^+^ deficiencies in diabetes, hepatic steatosis, and aging have historically used the NAD^+^ salvage pathway supplements: NAM or nicotinic acid (NA) [38,39]. Nicotinic acid can bind to the GPR109A receptor, a G protein-coupled receptor that binds NA resulting in severe flushing as a side effect, making it unfavorable to most patients [39]. Alternatively, nicotinamide riboside (NR) and NMN do not bind to the GPR109A receptor and are considered to have fewer unfavorable side effects [39]. Several studies have looked at NAM and NR as possible therapeutics for Alzheimer's disease, although none have used NMN. Liu et al. [40] administered NAM for 8 months in triple transgenic AD mice, finding NAM reduced beta amyloid (Aβ) and tau pathologies, elevated brain NAD^+^ levels, improved brain bioenergetics, and preserved mitochondrial functionality. In a separate study, Green et al. [41] treated four months old triple transgenic AD mice for 4 months, finding NAM treatment decreased tau levels and improved cognition. Similarly, when adult neurons isolated from triple transgenic AD mice aged 2 or 21 months were treated with NAM for 15 hours, NADH regenerating capacity was completely restored [42]. In a different AD disease mouse model, Gong et al. [43] treated Tg2576 animals with NR from 5–6 month of age until 10–11 months of age. NR treated mice had increased brain NAD^+^ levels, elevated Peroxisome proliferator-activated receptor gamma coactivator 1**-**alpha (PGC-1α), reduced Aβ, and reduced Beta site APP cleaving enzyme 1 (BACE1) [43]. Our present study is the first report using NMN in any AD mouse model, treating them temporally prior to amyloid deposition to focus on mitochondrial bioenergetics.

Sirtuins (SIRT 1–7) are class III histone deacetylases and NAD+ dependent enzymes [44]. Activation of SIRT1 mainly exerts neuroprotective actions [1] for example by deacetylating target proteins including PGC-1α, demonstrated to be deficient in human AD brain [45]. PGC-1α activation gives rise to mitochondrial biogenesis. In another study in APP/PS1 AD mice, overexpression of SIRT1 improved behavior and reduced Aβ [46]. In the present study, AD-Tg mice had significantly increased SIRT1 immunoreactivity compared to non-transgenic (NTG) mice that NMN treatment reduced (Figure 4) potentially preserving overall NAD^+^ pools for functional mitochondrial energetics.

Mitochondrial energetics in NMN treated animals could also be influenced by dynamic efficiency and the balance between fission and fusion. Mitochondrial fusion is regulated by the short and long isoforms of the protein Optic atrophy 1 (Opa1), as well as Mitofusin proteins (Mfn1 and Mfn2). Long and short OPA1 isoforms are both required for fusion. There is a reduction in fusion and an increase in fragmentation when the long OPA1 isoforms are converted to the short soluble OPA1 isoforms [47]. This can be the result of a reduction in mitochondrial membrane potential and can lead to mitophagy and cellular death [47]. Mitochondrial fission is regulated by Dynamin related protein Drp1, specifically by post-translational modifications of Drp1 [18]. Short round mitochondria occur more commonly with dysfunctional mitochondria resulting from treatment with a toxin or mtDNA depletion [48]. In this study we found a significant increase in P^616^-DRP1 immunoreactivity in AD-Tg mouse mitochondria compared to NTG and AD-Tg NMN treated animals. This would indicate AD-Tg mice might have more fission and fragmentation of mitochondria than the NTG mice. Furthermore, NMN reduced fission in the treated AD-Tg mice reverting P^616^-DRP1 levels to those of NTG animals.

To more clearly investigate mitochondrial morphology, CA1 hippocampal sections from CaMK2a-mito/eYFP mice were examined. These bigenic mice were previously determined to have functional mitochondrial bioenergetics similar to non-transgenic littermates [21]. NMN treated CaMK2a-mito/eYFP mice had longer mitochondria and reduced fragmentation, while vehicle treated mice had a greater proportion of spherical shaped mitochondria, possibly indicating more fission. There were no differences in total mitochondrial volume and the NMN treated samples had slightly fewer mitochondria, suggesting, NMN reduces fission or increases fusion. Taken together, NMN treatment decreased fission (P^616^-DRP1, AD mice) and reduced fragmentation (CaMK2a-mito/eYFP mice) suggesting a shift in dynamics from fission to fusion. This shift in dynamics could explain amelioration of mitochondrial bioenergetic deficits in the AD-Tg mice given NMN.

## Conclusions

We have established a potentially promising therapeutic protocol for the use of NMN as a viable supplement to reduce the cellular NAD^+^ deficits found in an AD mouse model. Administration of NMN improved mitochondrial bioenergetics in an AD-Tg AD mouse model as well as reduced mitochondrial fragmentation in mice possessing fluorescent proteins targeted to neuronal mitochondria without any observable negative side effects. The present study used young AD-Tg mice that had yet to form Aβ plaques given a low dose of NMN administered every other day for one month. It remains unclear if NMN prevented the mitochondrial deficits or remediated them. Reduction of full-length mutant APP following NMN treatment may also play a role in amelioration of mitochondrial bioenergetic function. Further studies need to be performed to examine potential benefit of NMN administered to older mice with progressive disease pathology. Furthermore, it is unknown whether a higher dose of NMN or longer duration of administration would be even more beneficial. By showing NMN can improve mitochondrial bioenergetics and dynamics in the mouse brain, we conclude that NMN has the potential to be a promising therapeutic for further testing on other neurological disease models with known NAD^+^ deficits.

## Abbreviations

Aβ: Beta amyloid
AD: Alzheimer’s disease
AD-Tg: APP_(swe)_/PS1_(ΔE9)_ transgenic
ALS: Amyotrophic lateral sclerosis
AMP: Adenosine monophosphate
APP: Amyloid precursor protein
APP_swe_: Amyloid precursor protein with the Swedish mutation
ATP: Adenosine triphosphate
BACE1: Beta site APP cleaving enzyme 1
CAMKII: Calcium/calmodulin-dependent kinase II
CD38: ADP-ribosyl cyclase
DRP1: Dynamin-related protein 1
EYFP: Enhanced yellow fluorescent protein
FCCP: Carbonyl cyanide *p*-(trifluoromethoxy)phenylhydrazone
GAPDH: Glyceraldehyde 3-phosphate dehydrogenase
HD: Huntington’s disease
IMM: Mitochondrial inner membrane
IMS: Inter-membrane space
MFN1: Mitofusin 1
MFN2: Mitofusin 2
MS: Multiple sclerosis
NA: Nicotinic acid
NAD: Nicotinamide adenine dinucleotide
NAM: Nicotinamide
Nampt: Nicotinamide phosphoribosyltransferase
NMN: Nicotinamide mononucleotide
NMNAT: Nicotinamide mononucleotide adenyltransferase
NR: Nicotinamide riboside
NTG: Non-transgenic
OCR: Oxygen consumption rates
OMM: Mitochondrial outer membrane
OPA1: Optic atrophy protein
PARP1: Poly (ADP-ribose) polymerase 1
PBS: Phosphate buffered saline
PBST: Phosphate buffered saline (PBS) with 0.1% tween-20
PCR: Polymerase chain reaction
PD: Parkinson’s disease
P^616^-DRP1: Phospho-DRP1
PGC1a: Peroxisome proliferator-activated receptor gamma coactivator 1**-**alpha
PS1: Mutant human presenilin 1
PS1_ΔE9_: Mutant human presenilin 1 with the delta E9
SIRT1: Sirtuin 1
TCA: Tricarboxylic acid
tTA: Tetracycline-controlled transactivator protein
TTA: Tetracycline transactivator
VDAC: Voltage dependent anion channel
